# The 41BB-agonist potentiates the therapeutic efficacy of a combined irreversible electroporation ablation treatment of lung cancer by promoting unexpected CD8^+^CD103^+^ cDC1 and tissue-resident memory T cell responses

**DOI:** 10.3389/fimmu.2025.1688281

**Published:** 2025-10-21

**Authors:** Chen Fang, Zhaojia Wu, Scot C. Leary, Yiling Bai, Michelle Yu, Nicolas Baniak, Shahid Ahmed, Gary Groot, Michael Moser, Wenjun Zhang, Bing Zhang, Junqiong Huang, Haitao Ma, Yu Feng, Jim Xiang

**Affiliations:** ^1^ Saskatoon Cancer Center, Saskatchewan Cancer Agency, Saskatoon, SK, Canada; ^2^ Department of Oncology, University of Saskatchewan, Saskatoon, SK, Canada; ^3^ Department of Thoracic Surgery, The First Affiliated Hospital of Soochow University, Suzhou, China; ^4^ Department of Biochemistry, Microbiology and Immunology, University of Saskatchewan, Saskatoon, SK, Canada; ^5^ Department of Pathology, University of Saskatchewan, Saskatoon, SK, Canada; ^6^ Department of Surgery, University of Saskatchewan, Saskatoon, SK, Canada; ^7^ Division of Bioengineering, University of Saskatchewan, Saskatoon, SK, Canada; ^8^ School of Mechatronic Engineering and Automation, Shanghai University, Shanghai, China; ^9^ Clinical Lab, Affiliated Hospital of Zunyi Medical University, Zunyi, China; ^10^ Department of Chest Surgery, Dushu Lake Hospital affiliated to Soochow University, Suzhou, China

**Keywords:** IRE-ablation, 41BB-agonist, TLR3/9 agonists, PD-1 blockade, CD8+ TRM cell, cDC1, lung cancer, tumor microenvironment

## Abstract

Irreversible electroporation (IRE) is a relatively new, non-thermal ablation technology for cancer treatment that requires further investigation to optimize its therapeutic efficacy. To improve IRE-ablation, we developed an IRE+Combo-treatment regimen that included the Combo adjuvants poly-I:C (pIC)/CpG, anti-PD-L1 antibody (PD-L1-Ab) and the 41BB-agonist, and investigated its anti-tumor immunity in a 3LL_OVA_ lung cancer model. We demonstrated that inclusion of the 41BB-agonist in the IRE+Combo-ablation stimulated a more efficient CD8^+^ T cell response (5.3%) than that observed in the absence of 41BB-agonist (3.0%) or upon IRE ablation alone (0.4%), leading to eradication of subcutaneous 3LL_OVA_ cancer in 75% of 3LL_OVA_-bearing mice. We further showed that the IRE+Combo-treatment regimen resulted in the eradication of both 3LL_OVA_ cancer and lung tumor metastases. Interestingly, our flow cytometry analyses argued that addition of the 41BB-agonist to the IRE+Combo-ablation stimulated a higher frequency of novel CD8^+^CD103^+^ conventional type-1 dendritic cells (cDC1) (14.4%) in tumor-drainage lymph-nodes (TDLNs) relative to control IRE+CpG/pIC/PD-L1-Ab- (7.5%) and IRE- (4.0%) treatment groups. This novel cDC1 subpopulation exhibited the most robust expression of DC maturation markers and costimulatory 41BBL and 41BB of all cDC1 subsets. The 41BB-agonist also stimulated a higher frequency of 41BB^+^CD103^+^TCF-1^+^ tissue-resident memory T (T_RM_) cells (14.5%) in TDLNs when compared with the two control (2.6% and 0.3%) treatment groups. Importantly, the IRE+Combo-treatment regimen was more efficient than the two control groups at converting the immunosuppressive tumor microenvironment (TME), an effect that was mitigated by reducing the frequency of inhibitory myeloid-derived suppressive cells while increasing that of immunogenic cDC1 and CD8^+^ T cells and rescuing T cell exhaustion. Taken together, our data establish that the 41BB-agonist potentiates the efficacy of IRE+Combo-therapy for lung cancer treatment by promoting unexpected cDC1 and T_RM_ cell responses, and emphasize the importance of targeting this promising molecular signal to improve current cancer IRE-ablation protocols.

## Introduction

Irreversible electroporation (IRE) is a new, non-thermal form of cancer ablation technology that delivers short bursts of current to induce irreversible, nano-sized holes in cell membranes, leading to broad tumor cell apoptosis (1, 2). Unlike thermal forms of ablation technologies like radiofrequency, high intensity focused ultrasound and microwaves that cause a “heat sink” effect and induce collateral damage to nearby tissue structures, IRE does not damage adjacent blood vessels, bile ducts and nerves or induce inflammation of surrounding non-malignant tissue, making it more suitable for ablation therapy of irresectable cancers (1, 2). IRE-induced apoptotic tumor cells leak a significant amount of immunogenic damage-associated molecular patterns (DAMPs) tumor antigens (TAs) and high mobility group protein B1 (HMGB1) along with chaperonins like heat shock protein 70 (HSP70) and calreticulin, which act collectively to stimulate dendritic cells (DCs) in IRE-ablated tumors (1–3). These DCs in turn phagocytose TA-expressing apoptotic tumor cell fragments and present these TAs to trigger TA-specific CD8^+^ T cell responses (4, 5). IRE-ablation therapy has been applied to various malignancies such as liver, pancreatic, breast, lung and prostate cancers (3). Clinical trial studies showed that IRE-ablation of cancer is safe and without serious, adverse side effects and is also associated with some, prolonged survival; as a whole, however, its therapeutic efficacy remains very poor (6, 7). Therefore, there is an urgent need to further improve the therapeutic efficacy of IRE-ablation of cancer.

Toll-like receptors (TLRs) recognizing conserved molecular patterns derived from pathogens initiate innate immune responses and enhance adaptive immunity (8). For example, the TLR3 agonist poly I:C (pIC) and TLR9 agonist CpG have been found to stimulate CD4^+^ type-1 helper T (Th1) and CD8^+^ cytotoxic T cell responses respectively (9, 10), and are commonly applied to improve immunotherapy (11, 12). Immune checkpoint inhibitor PD-1 blockade using anti-PD-L1 antibody (PD-L1-Ab) triggers potent antitumor immunity or rescues T cell exhaustion by blocking the inhibitory PD-1/PD-L1 pathway in CD8^+^PD-1^+^ T cells (13, 14), and has also commonly been used in clinical cancer immunotherapy (15). Finally, a 41BBL triggered T cell 41BB signal has been shown to promote CD8^+^ tissue-resident memory T (T_RM_) cell responses (16), and co-stimulation with a 41BB-agonist like anti-41BB Ab promotes CTL expansion, cytotoxicity and survival in addition to converting CTL exhaustion, thus potentiating cancer immunotherapy (17–22).

We previously developed a combinatorial IRE treatment protocol that combined IRE-ablation with administration of immune adjuvants (CpG, pIC and PD-1-blockade) and investigated its therapeutic efficacy in an EG7 lymphoma model (23). We found that the combined IRE+CpG/pIC/PD-L1-Ab ablation cooperatively induced potent CD8^+^ T cell immunity, which led to complete eradication of both the EG7 lymphoma and lung metastases (23). We then assessed the effectiveness of this approach for the treatment of solid cancers in a 3LL_OVA_ lung cancer (50 mm^3^) model and found that it failed to eradicate 3LL_OVA_, though it significantly inhibited tumor growth (23). Therefore, to further improve upon its therapeutic efficacy in solid cancers we developed a novel IRE+Combo treatment protocol that combined IRE+CpG/pIC/PD-L1-Ab with the 41BB-agonist and assessed its stimulatory effects on T cell immunity and its therapeutic efficacy against lung cancer. We demonstrate herein that this new IRE+Combo ablation regimen stimulated potent CD8^+^ T cell responses in mouse peripheral blood. More specifically, we provide the first evidence that inclusion of the 41BB-agonist in the IRE+Combo regimen promoted unexpected responses from CD8^+^CD103^+^TCF-1^+^41BB^+^ tissue-resident memory T (T_RM_) cells and conventional type-1 DC (cDC1) subsets in tumor-drainage lymph nodes (TDLNs). The cDC1 subsets were represented by CD8^+^ and CD103^+^ cDC1s as well as an unexpected CD8^+^CD103^+^41BB^+^ cDC1 cohort which exhibited greater expression of the DC maturation markers CD54, CD80 and MHC II and the DC co-stimulatory 41BBL and T cell co-stimulatory 41BB molecules. Collectively, the above immune responses led to the eradication of 75% of subcutaneous (s.c.) 3LL_OVA_ cancers and the complete elimination of lung tumor metastases. Importantly, we also demonstrated that the new IRE+Combo treatment regimen more efficiently converted the immunosuppressive tumor microenvironment (TME) than the IRE+CpG/pIC/PD-L1-Ab treatment lacking the 41BB-agonist by reducing the frequency of immunosuppressive myeloid-derived suppressive cells (MDSC) while increasing that of immunogenic cDC1 and CD8^+^ T cells and rescuing CD8^+^ T cell exhaustion.

## Materials and methods

### Reagents, cell lines and mice

A phycoerythrin (PE)-conjugated H-2K^b^/OVA_257–264_ tetramer (PE-tetramer) was obtained from the Fred Hutchinson Cancer Research Center (Seattle, WA). A fluorescein isothiocyanate (FITC)-conjugated anti-CD8 antibody (Ab) was obtained from Bio-Rad (Hercules, CA, USA). The following Abs and reagents were obtained from BioLegend (San Diego, CA, USA); PE/Cy5-conjugated anti-CD8, PE-conjugated anti-CD45.1, FITC-conjugated anti-CD45.1, APC/Cy7-conjugated anti-CD3, PE-conjugated anti-CD103, BV421-conjugated anti-CD103, APC-conjugated anti-CD11b, PE-Cy5-conjugated anti-CD11c, APC/Cy7-conjugated anti-I-A/I-E (MHC II), BV421-conjugated anti-Ly6G, PE/Cy5-conjugated anti-Gr1, BV421-conjugated anti-CD44, BV421-conjugated anti-CD62L, APC-conjugated anti-TCF1, Alex Flour-647-conjugated anti-PD-L1, PE-conjugated anti-PD-L1, biotin-conjugated anti-LAG3, anti-PE-avidin, FITC-avidin, anti-rabbit-PE and anti-rabbit-FITC Abs and the Zombie Aqua Fixable Viability Kit. The following Abs were obtained from BD Biosciences (San Jose, CA, USA); PE-conjugated anti-TOX, biotin-conjugated anti-I-A/I-E (MHC II), biotin-conjugated anti-CD80, biotin-conjugated anti-CD54 and biotin-conjugated anti-PD-L1 Abs. The anti-HSP-70, anti-HMGB1, anti-calreticulin Abs were obtained from Cell Signaling Technology (Danvers, MA, USA). CpG oligodeoxynucleotides 1826 (CpG ODN 1826) and poly:IC (pIC) were obtained from Invitrogen Inc (San Diego, CA, USA). The Cytofix/Cytoperm kit and lysis buffer were purchased from BD Biosciences (Franklin Lakes, NJ). Anti-PD-L1 and anti-41BB Abs for *in vivo* use were obtained from BioXCell (Lebanon, NH, USA) and InvivoGene (San Diego, CA, USA), respectively. The mouse lung cancer cell line 3LL_OVA_ was generated in our laboratory by transfecting the OVA transgene into the 3LL lung cancer cell line obtained from the American Type Culture Collection (ATCC; Rockville, MD, USA). The highly lung metastatic OVA-expressing BL6-10_OVA_ (BL_OVA_) melanoma cell line was previously generated in our lab (23, 24). 3LL_OVA_ and BL_OVA_ cells were maintained in RPMI medium (Life Technologies, Carlsbad, CA, USA) supplemented with 10% fetal calf serum (FCS) and G418 (0.5 mg/mL; Life Technologies). Female C57BL/6 (B6, CD45.2) and B6.1 (CD45.1) mice were purchased from the Jackson Laboratory (Bar Harbor, ME, USA). All animal experiments were approved by the Animal Research Ethics Board, University of Saskatchewan (Protocol# 20160056).

### Analysis of *in vitro* electroporation-induced tumor cell apoptosis

Mouse lung cancer 3LL_OVA_ cells were electroporated using a Bio-Electroporator (Bio-Rad) with 0V (control), 600V, 1200V and 1800V to induce tumor cell apoptosis. Cells were stained with FITC-labeled Annexin-V and propidium iodide (PI) and analyzed by flow cytometry to quantify the percentage of electroporation-induced Annexin-V^+^PI^+^ apoptotic tumor cells and the relative expression of immunogenic macromolecules like HMGB1, HSP70 and calreticulin (23, 24). To assess the immunogenicity of apoptotic tumor cells, apoptotic 3LL_OVA_ cells (1×10^6^ cells/mouse) derived from electroporation with 1200V, which is the same voltage used for *in vivo* IRE, and 600V as a control were i.v. injected into C57BL/6 mice (8 mice/group). Mouse blood samples were then stained with FITC-anti-CD8 Ab (FITC-CD8) and PE-tetramer 8 days post-injection for flow cytometry quantitation of OVA-specific CD8^+^ T cell responses (23, 24).

### Preparation and characterization of DCs that phagocytose apoptotic tumor cells

B6 mouse bone marrow-derived DCs were prepared as previously described (24). 3LL_OVA_ cells were labeled with carboxyfluorescein succinimidyl ester (CFSE) (4μM), followed by a 1200V treatment. DCs were then cultured with apoptotic CFSE (green)-3LL_OVA_ cells at a ratio of 1:2 overnight to promote phagocytosis of apoptotic 3LL_OVA_ cells (DC/3LL_OVA_), stained with APC-anti-CD11c Ab (red) and DAPI (blue) and analyzed by flow cytometry and confocal microscopy (24). To assess its stimulatory effect, C57BL/6 mice (8 mice/group) were i.v. immunized with DC/3LL_OVA_ (1×10^6^ cells/mouse) and blood samples were taken 8 days later and stained with FITC-CD8 Ab and PE-tetramer for flow cytometry analysis (24).

### Treatment of 3LL_OVA_ lung cancer with IRE, IRE+CpG/pIC/PD-L1-Ab and IRE+Combo

3LL_OVA_ cells (1×10^6^ cells/mouse) were injected subcutaneously (s.c.) into the right flank of C57BL/6 mice. Tumor growth was monitored daily using a digital caliper, and tumor volumes were calculated using the formula A/2×B^2^ where A and B are the long and short dimensions of the tumor, respectively (23). When tumors reached 50 mm^3^ (4.5-5.0 mm in diameter), tumor-bearing mice (8 mice/group) were treated with various IRE ablation protocols. These included IRE+CpG/pIC/PD-L1/41BB Abs (i.e. IRE+Combo treatment regimen) and various control treatment protocols; (i) control (without any treatment), (ii) IRE alone, (iii) IRE+CpG/pIC, (iv) IRE+PD-L1 Ab, (v) IRE + 41BB Ab, (vi) IRE+CpG/pIC/PD-L1-Ab and (vii) CpG/pIC/PD-L1/41BB-Abs (Combo) alone. Briefly, mice were intratumorally (i.t.) injected with a mixture of CpG (30μg) and pIC (30μg) in a total of 15μL PBS into three different points (5μL/point) in peripheral tumor tissues prior to IRE-ablation at day 0 (23) and intraperitoneally (i.p.) injected with anti-PD-L1 (150µg/mouse) and anti-41BB (100µg/mouse) Abs one day prior to IRE ablation (day -1) and then once every two days (day 1, 3, 5, 7) for a total of five injections (23). The IRE-ablation was performed at day 0. IRE parameters were voltage, 1,200V/cm; pulse duration, 90µs; pulse repetition frequency, 1 Hz; and, number of repeated pulses, 100 (23). The electrode was then rotated 90° and the process repeated as we have previously described (23). To evaluate OVA-specific CD8^+^ T cell responses, mouse peripheral blood samples were stained 8 days post-ablation with FITC-CD8 Ab and PE-tetramer for flow cytometry analysis (23). Tumor growth or regression was monitored daily. For ethical reasons, mice carrying tumors of approximately 500 mm^3^ (9–10 mm) were sacrificed and classified as dead (23, 24).

### Measurement of cancer metastatic colonies on the surface of lung tissues in IRE+Combo-treated mice

3LL_OVA_ cells (1×10^6^ cells/mouse) were s.c. injected into the right flank of B6 mice (8 mice/group) (23). Seven days later, tumor-bearing mice and control mice without any treatment were i.v. injected with BL_OVA_ cells (0.5×10^6^ cells/mouse) (23). After another 7 days, IRE+Combo treatment was performed in mice bearing s.c. 3LL_OVA_ tumors (~50 mm^3^). IRE+Combo-treated and control mice were euthanized 14 days later, and lung tissues were collected (23). Black tumor colonies on the surface of lung tissue were counted and confirmed by histopathological examination (23).

### Analysis of single cell suspensions derived from TDLNs

TDLNs were harvested from mice (5 mice/group) treated with IRE+Combo, IRE+CpG/pIC/PD-L1-Ab and IRE only 7–9 days after treatment. Single cell suspensions were prepared by passing lymphoid tissue through a 40 µm strainer using syringe trituration (23), and stained with Abs against cell surface CD11c, CD8, CD103, co-stimulatory 41BB and the DC maturation markers MHC II, CD54 and CD80 for analysis of cDC1 subsets. Samples were also stained with Abs against cell surface CD3, CD8, CD44, CD62L, CD69 and CD103 for analysis of CD8^+^ T_RM_ cells. To analyze the expression of intracellular TCF1 in CD8^+^ T_RM_ cells, single cell suspensions were first permeabilized using the Cytofix/Cytoperm kit (BD Biosciences) and then stained with anti-TCF1 Ab. All single cell suspensions were then analyzed by flow cytometry with a progressive gating strategy (23).

### Flow cytometric analysis of tumor-infiltrating immune cell profiles

B6.1 (CD45.1^+^) mice (5 mice/group) were inoculated with 3LL_OVA_ cells to evaluate immune cell profiling in tumors. Flow cytometry was used to differentiate between CD45.1^+^ mouse immune cells and CD45.2^+^ 3LL_OVA_ tumor cells in single cell suspensions obtained from tumors treated with IRE alone, IRE+CpG/pIC+PD-L1 or IRE+Combo. Tumor tissue samples were cut into 1 mm^3^ pieces and incubated in serum-free RPMI medium containing collagenase IV (1mg/ml) and DNase I (0.2mg/ml) for 30 minutes at 37 °C, as we have previously described (23). The digested tissue was then ground with a syringe and passed through a 40 μm filter. Single cell suspensions were incubated with red blood cell lysis buffer (0.84% Tris-ammonium chloride) for 5 min to remove erythrocytes, washed and resuspended in PBS supplemented with 2% FCS and 0.1% sodium azide. Zombie Aqua Fixable Viability dye was then used to distinguish live from dead cells before staining with a combination of fluorescently labeled antibodies to distinguish different immune cell populations such as cDC1, MDSCs and CD8^+^ T cells (23). Flow cytometry analysis involved initial gating for CD45.1^+^ immune cell populations to distinguish them from CD45.2^+^ 3LL_OVA_ tumor cells, followed by subsequent gating for various immune cell populations based on specific cellular markers (23). For instance, the DC population was gated as CD11c^+^ and further analyzed to quantify the percentage of CD8^+^CD103^+^ cDC1 subpopulations. The monocyte population was gated as CD45.1^+^ and further analyzed to quantify the percentage of CD11b^+^Gr1^+^Ly6G^+^ MDSCs, CD8^+^CD103^+^ cDC1 and CD8^+^ T cells. For flow cytometry analysis of intracellular staining, cells were first stained for surface markers then fixed and permeabilized using a Cytofix/Cytoperm kit (BD Biosciences) and stained with Abs against intracellular markers such as IDO, arginase 1, TOX and TCF1 (23). All flow cytometry data were acquired with a CytoFLEX cytometer (Beckman Coulter Inc.) and analyzed using FlowJo (10.4.0) software (FlowJo, LLC, Ashland, OR) (23).

### Histopathological analysis

Tumor tissues were fixed with 10% formalin and then embedded in paraffin. Paraffin-embedded tissue blocks were sectioned into 6 μm slices and affixed to slides, stained with hematoxylin-eosin, and visualized by microscopy at 50× and 200× magnification (23, 24).

### Statistical analysis

Tumor growth curves were analyzed using the *log-rank* test (23, 24). For comparisons between two experimental groups, a two-tailed Student’s *t*-test was applied. Multiple comparisons were conducted using a one-way ANOVA followed by a *Tukey’s* test (23, 24). Statistical values were defined as p<0.05, significant; p<0.01, very significant. Results are presented as the mean ± standard error of the mean (SEM) (23, 24).

## Results

### Tumor cells undergoing *in vitro* IRE-induced apoptosis are immunogenic

**Figure 1:**
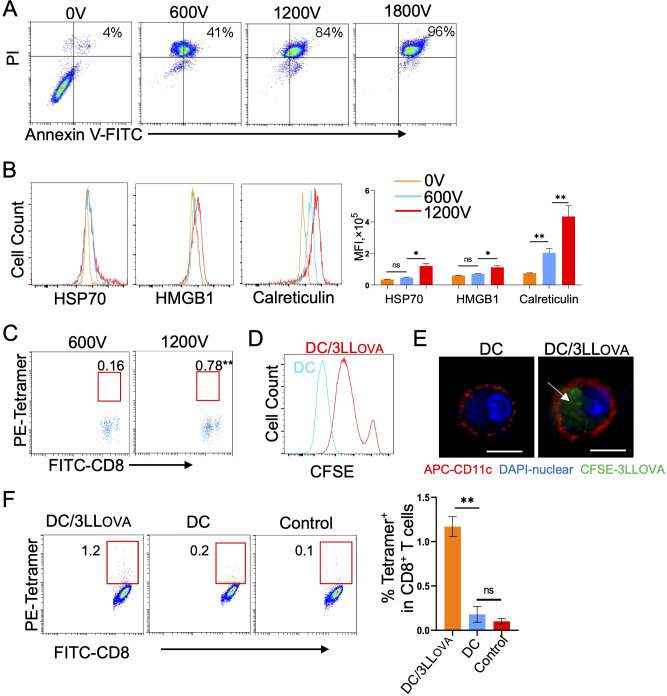
IRE (1200V)-induced apoptotic tumor cells are immunogenic. **(A)** 3LL_OVA_ cells treated with different voltages (0V, 600V, 1200V, and 1800V groups) using a BioRad electroporator were stained with FITC-labeled anti-Annexin V antibody (green) and PI (red), and then the percentage of apoptotic cells expressing both Annexin V and PI was measured in each group by flow cytometry. **(B)** The 0V-, 600V- and 1200V-treated 3LL_OVA_ cells were stained with anti-HSP70 Ab, anti-HMGB1 Ab and anti-Calreticulin Ab, respectively, and then analyzed by flow cytometry. The values represent the mean fluorescence intensity (MFI) of apoptotic cells expressing HSP70, HMGB1 and calreticulin. **(C)** Blood samples derived from mice immunized with 600V- or 1200V-treated apoptotic 3LL_OVA_ cells were stained with OVA-specific PE-Tetramer and FITC-labeled anti-CD8 antibody and analyzed by flow cytometry. The value in each panel represents the percentage of OVA-specific CD8^+^ T cells in the total CD8^+^ T cell population. **(D)** Flow cytometry histogram showing the fluorescence intensity of control DCs (blue line) and DCs containing phagocytosed CFSE-labeled 1200V-treated 3LL_OVA_ cell fragments (red line). **(E)** Representative confocal images showing CFSE (green)-labeled 1200V-treated 3LL_OVA_ cell fragments phagocytosed into the cytoplasm of PE (red)-labeled CD11c-positive membrane of DC with DAPI (blue)-stained DC nuclei. The white arrow, the phagocytosed apoptotic 3LL_OVA_ cell fragments. **(F)** Blood samples derived from mice immunized with DCs that phagocytosed 1,200-treated 3LL_OVA_ cells (DC/3LL_OVA_) or regular DCs (DC) or blood samples derived from naïve mice (control) were stained with OVA-specific PE-Tetramer and a FITC-labeled anti-CD8 antibody and analyzed by flow cytometry. The value in each panel represents the percentage of OVA-specific CD8^+^ T cells among the total CD8^+^ T cell population. *P < 0.05, **P < 0.01 by two-tailed Student *t*-test.

To assess the effect of different volts on tumor cell death, we treated OVA-expressing 3LL_OVA_ lung cancer cells *in vitro* with 0V, 600V, 1200V and 1800V using a BioRad electroporator. Treated cells were then stained with Annexin-V and PI, early and late apoptotic markers respectively, and analyzed by flow cytometry. These analyses demonstrated that the abundance of Annexin-V^+^/PI^+^ late stage, apoptotic cells was positively correlated with the electroporation voltage (**Figure 1A**). We observed that with 1200V, which is the same voltage used for *in vivo* IRE, 84% of tumor cells were late-stage apoptotic (**Figure 1A**). Next, we measured expression of immunogenic proteins such as HMGB1, HSP70 and calreticulin in 1200V-treated apoptotic tumor cells by flow cytometry and demonstrated that they were all upregulated when compared to untreated or 600V-treated cells (**Figure 1B**). To further assess the immunogenicity of apoptotic tumor cells, we i.v. immunized C57BL/6 mice with the 1200V-treated tumor cells. Mouse peripheral blood samples then were stained with FITC-anti-CD8 Ab and PE-Tetramer and analyzed by flow cytometry eight days post-immunization. These analyses revealed that 1200V-treated tumor cells stimulated stronger OVA-specific CD8^+^ T cell responses (0.78%) than 600V-treated ones (0.16%) (**Figure 1C**), indicating that *in vitro* 1200V-treated apoptotic tumor cells are indeed more immunogenic.

### DCs phagocytose 1200V-treated tumor cells and stimulate CD8^+^ T cell responses

DCs were first prepared by culturing mouse bone marrow cells *in vitro* for two days in the presence of GM-CSF, followed by an additional day in the presence of GM-CSF and IL-4 (24). DCs were then co-cultured overnight with CFSE-labeled 1200V-treated 3LL_OVA_ tumor cells and analyzed by flow cytometry and confocal microscopy. Both approaches confirmed that DCs phagocytose CFSE-labeled apoptotic 3LL_OVA_ cells or cellular fragments (i.e. DC/3LL_OVA_) (**Figures 1D, E**). Consistent with this observation, we found by flow cytometry that i.v. immunization of mice with DC/3LL_OVA_ cells stimulated moderate CD8^+^ T cell responses (1.2%) 8 days post-injection (**Figure 1F**). These data indicate that DCs are capable of stimulating OVA-specific CD8^+^ T cell responses once they phagocytose 1200V-induced apoptotic 3LL_OVA_ cells.

### *In vivo* IRE-ablation induces significant tumor cell apoptosis and weak OVA-specific CD8^+^ T cell responses

**Figure 2:**
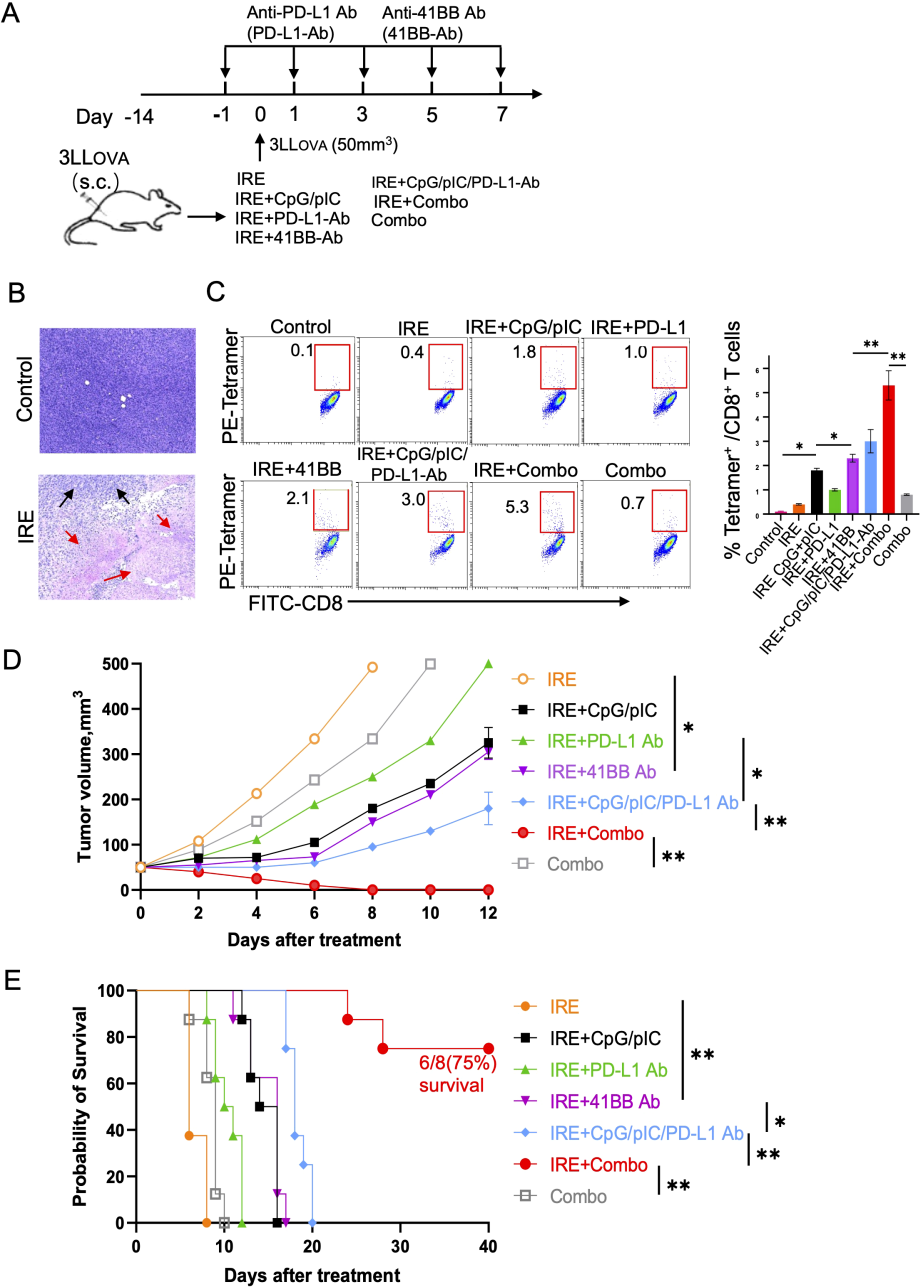
IRE+Combo ablation induces potent anti-tumor immunity. **(A)** A diagram summarizing the experimental design of the IRE treatment groups. **(B)** Tumor apoptosis was detected by H&E staining of tissue sections from tumors collected from mice with (IRE) or without (control) IRE treatment. Black arrow, tumor; red arrows, tumor apoptosis. **(C)** Blood samples derived from all seven indicated groups of mice with treatment and one group of untreated, control mice were stained with OVA-specific PE-Tetramer and a FITC-labeled anti-CD8 antibody and analyzed by flow cytometry. The value in each panel represents the percentage of OVA-specific CD8^+^ T cells among the total CD8^+^ T cell population (right). **(D)** Tumor-bearing mice were treated with each of the seven treated groups of mice and then monitored for tumor growth or regression. Tumor growth curves were analyzed by two-way ANOVA with a *Tukey*’s test. *P < 0.05, **P < 0.01. **(E)** Tumor-bearing mice were also monitored for survival post IRE ablation by using a *log-rank* test. *P < 0.05, **P < 0.01. Tumor growth, mouse survival and flow cytometric plots represent one of two independent experiments.

To assess IRE-induced tumor cell apoptosis, 3LL_OVA_-bearing C57BL/6 mice were subjected to IRE-ablation when tumors reached 4.5–5 mm in diameter (50 mm^3^) using the previously described parameters (voltage: 1,200 V/cm; pulse duration: 90μs; pulse repetition frequency: 1 Hz; number of repetition pulses: 100) applied to cancer patients in the clinic (23) (**Figure 2A**). We then conducted histopathological examination of tumor tissue sections and measured CD8^+^ T cell responses by flow cytometry 3 and 8 days post-ablation, respectively (**Figure 2A**). These analyses revealed that while IRE-ablation induced a large central region of tumor cell apoptosis without affecting the remaining, peripheral tumor tissues (**Figure 2B**), it was only able to stimulate weak OVA-specific CD8^+^ T cell responses (0.4%) (**Figure 2C**).

### The IRE+Combo treatment regimen synergistically stimulates potent CD8^+^ T cell responses and leads to the eradication of lung cancer

To assess whether pIC, CpG, CpG/pIC, PD-L1 Ab, 41BB Ab, CpG/pIC/PD-L1-Ab and Combo (CpG/pIC/PD-L1/41BB Abs) are capable of enhancing OVA-specific CD8^+^ T cell responses and inhibiting 3LL_OVA_ tumor growth, we subjected our 3LL_OVA_ lung cancer model to these various treatment regimens (**Figure 2A**). We found that IRE+CpG/pIC and IRE + 41BB Ab stimulated comparable CD8^+^ T cell responses (1.8% vs 2.1%), which were stronger than those mediated by the IRE+PD-L1 Ab treatment (1.0%) (**Figure 2C**). The IRE+CpG/pIC/PD-L1-Ab treatment which combined these various arms stimulated stronger 3.0% CD8^+^ T cell responses (**Figure 2C**), showing a cooperative effect of targeting the TLR3/9 agonists and PD-1 blockade in CD8^+^ T cell immunity. Interestingly, the complete IRE+Combo treatment regimen acted synergistically to stimulate the most potent CD8^+^ T cell responses (5.3%), especially in light of the fact each arm of this combinatorial treatment elicited very modest CD8^+^ T cell responses in isolation (IRE-induced, 0.4%; Combo-triggered, 0.8%) (**Figure 2C**). To investigate their therapeutic effects, we performed the same ablation treatments and then monitored tumor growth daily using a digital caliper (23, 24). We found that the therapeutic effects of the various treatment groups on tumor growth inhibition and mouse survival displayed similar trends (**Figures 2D, E**) that mirrored the magnitude of CD8^+^ T cell responses (**Figure 2C**). Briefly, IRE- or Combo alone-treated mice showed minimal inhibition of tumor growth, and all animals died within 10 days of treatment (**Figures 2D, E**). IRE+CpG/pIC and IRE + 41BB Ab treatments inhibited tumor growth to the same extent, and all mice died roughly 15 days post-treatment (**Figures 2D, E**). The IRE+CpG/pIC/PD-L1-Ab ablation which we previously demonstrated was able to eradicate EG7 lymphoma (23) failed to eliminate 3LL_OVA_ lung cancer, though tumor growth was significantly inhibited and mouse survival was markedly prolonged (**Figures 2D, E**). Importantly, the addition of the 41BB-agonist to the IRE+CpG/pIC/PD-L1- Ab treatment to yield the IRE+Combo regimen completely eradicated s.c. 3LL_OVA_ cancer in 75% of treated mice (**Figures 2D, E**). These data collectively indicate that the 41BB-agonist plays a critical role in promoting the therapeutic efficacy of this combinatorial IRE+Combo ablation treatment.

### Ablation of local cancer by IRE+Combo treatment induces eradication of lung tumor metastases

**Figure 3:**
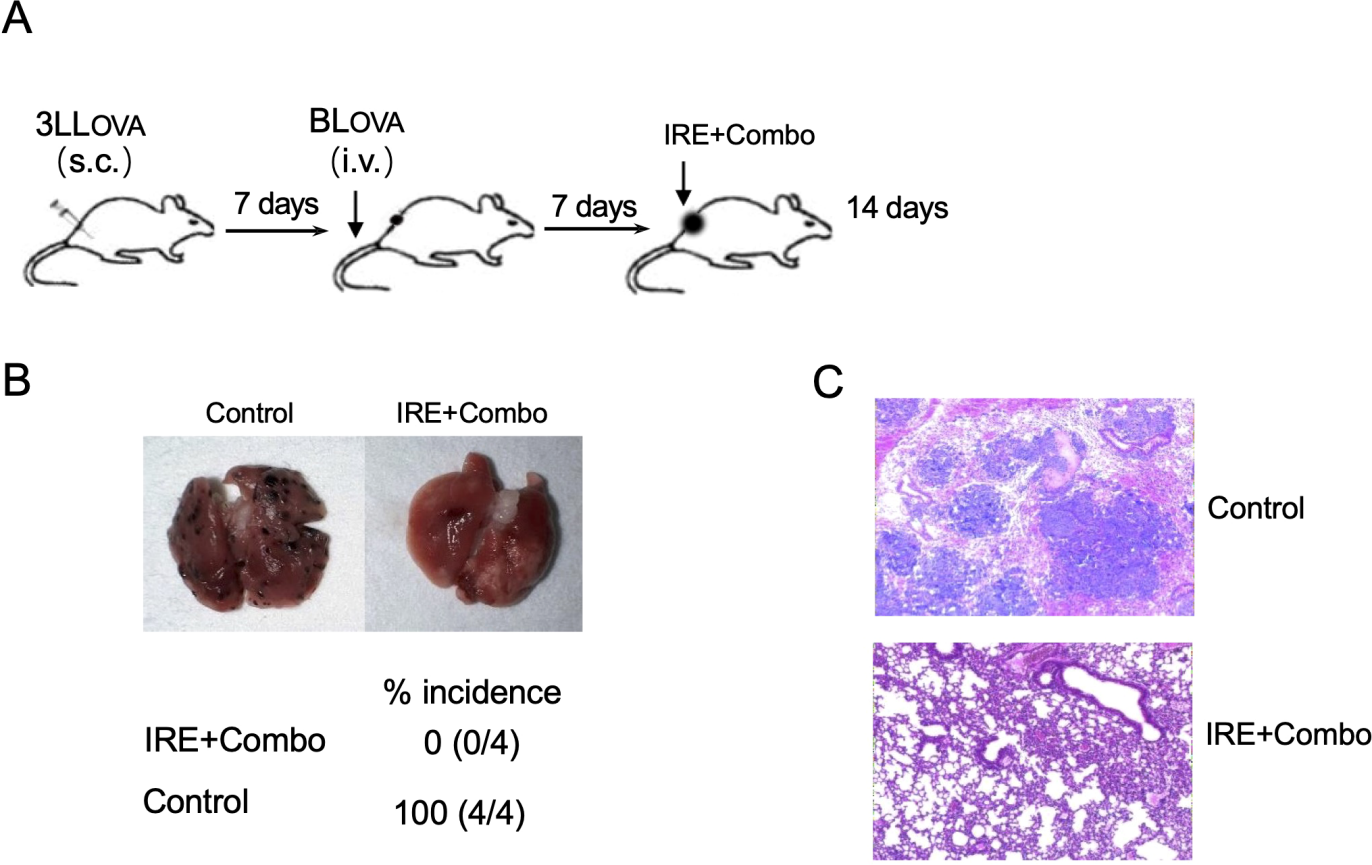
IRE+Combo ablation of primary tumors induces eradication of lung tumor metastasis. **(A)** Schematic diagram of the experimental design for assessing the anti-metastatic activity derived from IRE+Combo treatment of primary tumors as well as mice without any treatment as a control. Mice bearing small, 7-day 3LL_OVA_ tumors were further i.v. injected with highly metastatic BL_OVA_ melanoma cells. After another 7 days, IRE+Combo treatment was performed in mice bearing primary 3LL_OVA_ tumors as well as 7-day lung BL_OVA_ metastases. Mice were euthanized 14 days after IRE ablation, and lung tissues were collected. **(B)** One representative lung out of four is shown (upper). Black metastatic BL_OVA_ melanoma colonies in lungs were counted. The average numbers of lung metastatic BL_OVA_ colonies and the percentage of incidence of mouse lung metastasis for each group was also shown (lower). **(C)** Representative micrographs of H&E-stained lung tissue sections collected from each group. One representative experiment out of two independent experiments is shown.

To investigate if IRE+Combo ablation of local s.c. 3LL_OVA_ tumors also affects lung tumor metastases, we i.v. injected highly lung metastatic OVA-expressing BL_OVA_ melanoma cells into mice bearing 7 day old s.c. lung cancers or naïve, control animals (**Figure 3A**). One week after melanoma cell injection which coincided with s.c. 3LL_OVA_ tumors reaching 50 mm^3^, we performed IRE+Combo ablation in local 3LL_OVA_ tumors as previously described (23). We then collected mouse lung tissue two weeks later for visual detection of black melanoma metastatic colonies on the tissue surface, and histopathological analysis of tissue sections (23). These analyses demonstrated that a large number of black BL_OVA_ melanoma colonies were present in the lungs of control mice while none were detected in IRE+Combo-ablated mouse lungs (**Figures 3B, C**). Our data further argued that inclusion of the 41BB-agonist is critical to the eradication of both local s.c. 3LL_OVA_ cancer and lung BL_OVA_ tumor metastases.

### Inclusion of the 41BB-agonist in IRE+Combo ablation stimulates potent 41BB^+^CD8^+^CD103^+^ cDC1 maturation and expansion in TDLNs

**Figure 4:**
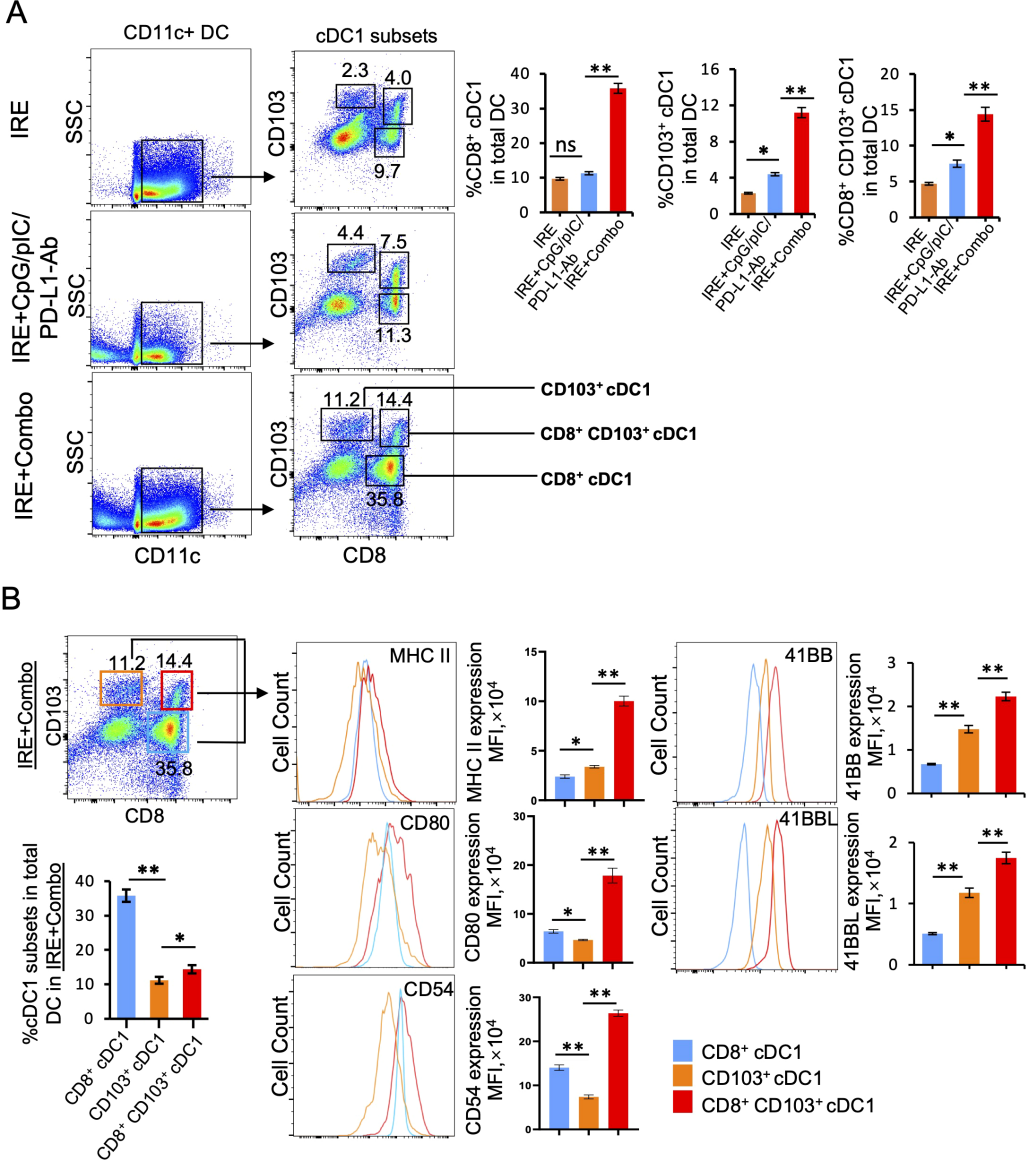
The 41BB agonist in IRE+Combo stimulates potent cDC1 subsets (CD8^+^, CD103^+^ and CD8^+^CD103^+^ cDC1) expansion and maturation in TDLNs (Part 1). Single-cell suspensions prepared from the TDLNs 8 days after three treatment groups IRE, IRE+CpG/pIC/PD-L1-Ab and IRE+Combo. Cell samples were stained with a cocktail of Abs (as described in the Methods) to measure the percentage of different cDC1 subsets and analyze expression of MHC II, CD54, CD80 and 41BB by flow cytometry, respectively. **(A)** Flow cytometry plots and bars showed quantitative measurement of the percentage of CD8^+^ cDC1, CD103^+^ cDC1 and CD8^+^CD103^+^ cDC1 cells by analysis of gated CD11c^+^ DCs in three treatment groups. **(B)** Flow cytometry plots and bars showed quantitative measurement of the percentage of CD8^+^ cDC1, CD103^+^ cDC1 and CD8^+^CD103^+^ cDC1 cells in the IRE+Combo group, followed by quantitative measurement of expression of MHC II, CD54, CD80 and 41BB in three cDC1 subsets. The values of the MFI of MHC II, CD54, CD80 and 41BB expression in the IRE+Combo treatment group were shown in flow cytometry histogram sets. *P < 0.05, **P < 0.01 by two-tailed Student *t*-test. The above data represent one of two independent experiments.

TDLNs are lymphoid organs where early anti-tumor immunity is initiated through CD11c^+^ DC-induced stimulation of CD8^+^ effector T (T_E_) cell responses (25). cDC1 is a superior DC subset capable of cross-presenting antigen and potently stimulating anti-tumor CD8^+^ T cell responses in both TDLNs and tumors (26, 27). In mice, cDC1 cells encompass the lymphoid tissue-resident CD8^+^ cDC1 and the non-lymphoid, tissue-resident CD103^+^ cDC1 subsets (28). Recently, however, it has been reported that CD103^+^ cDC1 can also migrate to lymph nodes (25, 29–31). To evaluate the ability of the IRE, IRE+CpG/pIC/PD-L1 Ab and IRE+Combo treatment regimens to trigger cDC1 responses, single cell suspensions were prepared from TDLNs 8 days post-treatment and analyzed by flow cytometry. Flow cytometry plots show quantitative measurement of the percentage of different cDC1 subsets within the total CD11c^+^ DC cell population, while histograms display the relative cell surface expression of the cDC1 maturation markers MHC II, CD80 and CD54 as well as the T cell co-stimulatory molecule 41BB. We found that IRE, IRE+CpG/pIC/PD-L1-Ab and IRE+Combo induced both CD8^+^ and CD103^+^ cDC1 responses but also led to the appearance of an unexpected CD8^+^CD103^+^ cDC1 cell population that was significantly expanded in mouse TDLNs in this therapeutic context (**Figure 4A**). However, all three CD8^+^, CD103^+^ and CD8^+^CD103^+^ cDC1 subsets (35.8%, 11.2% and 14.4% in total CD11c^+^ DCs) were found to be more abundant in IRE+Combo-ablated mouse TDLNs compared to IRE+CpG/pIC/PD-L1-Ab-ablated (11.3%, 4.4% and 7.5%) and IRE-ablated (9.7%, 2.3% and 4.0%) mouse TDLNs (**Figure 4A**), indicating that inclusion of the 41BB-agonist in IRE+Combo promotes more potent responses from all three localized cDC1 subsets.

**Figure 5:**
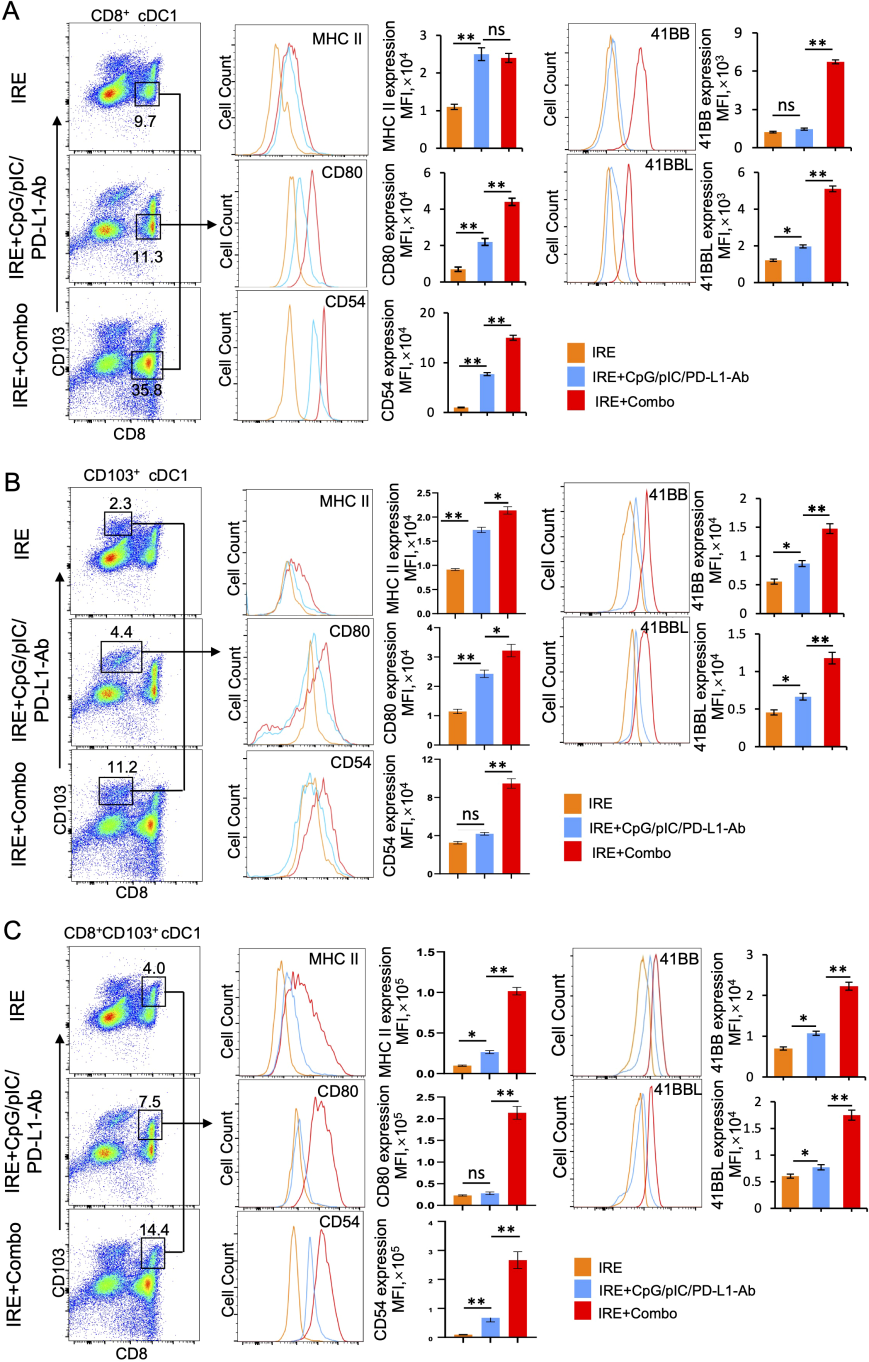
The 41BB agonist in IRE+Combo stimulates potent cDC1 subsets (CD8^+^, CD103^+^ and CD8^+^CD103^+^ cDC1) expansion and maturation in TDLNs (Part 2). Single-cell suspensions prepared and stained as described in **Figure 4**. The expression of MHC II, CD54, CD80 and 41BB in **(A)** CD8^+^ **(B)** CD103^+^ and **(C)** CD8^+^CD103^+^ cDC1 cells in three treatment groups were analyzed by flow cytometry, respectively. The values of the mean fluorescence intensity (MFI) of MHC II, CD54, CD80 and 41BB expression for each group were shown in flow cytometry histogram sets. The values of the MFI of MHC II, CD54, CD80 and 41BB expression were shown in flow cytometry histogram sets. *P < 0.05, **P < 0.01 by two-tailed Student *t*-test. The above data represent one of two independent experiments.

It has been reported that the co-stimulatory molecules CD54, CD80 and MHC II are DC maturation markers that potently stimulate CD8^+^ T cell responses (23, 24). DCs also express the DC co-stimulatory 41BBL and the T cell co-stimulatory 41BB molecules to promote cell survival (32, 33). Therefore, to assess whether inclusion of the 41BB-agonist in the IRE+Combo treatment regimen affects the maturation of cDC1 subsets, we used flow cytometry to measure the expression of these five molecules on cDC1 subsets derived from TDLNs of mice treated with IRE+Combo, IRE+CpG/pIC/PD-L1-Ab or IRE alone. Our representative flow cytometry histograms show the relative signal intensity of DC maturation markers and the co-stimulatory 41BB molecule on gated CD8^+^, CD103^+^ and CD8^+^CD103^+^ cDC1 cells for all three treatment groups. We found that the IRE+Combo ablation regimen stimulated more significant up-regulation of CD54, CD80 and MHC II as well as the co-stimulatory 41BB and 41BBL molecules on cDC1 subsets, except for MHC II expression in CD8^+^ cDC1 cells (**Figures 5A, B, C**). While the frequency of CD8^+^ cDC1 (35.8%) was roughly 2.5 fold higher than that of CD103^+^ and CD8^+^CD103^+^ cDC1 (11.2% and 14.4%) in TDLNs isolated from the IRE+Combo treatment group (**Figure 4B**), the CD8^+^CD103^+^ cDC1 subset displayed proportionally higher expression of all five molecules of interest (**Figure 4B**). Our data thus indicate that the 41BB-agonist in the IRE+Combo treatment stimulates potent responses from the CD8^+^CD103^+^ cDC1 subset, which displayed the highest expression of DC maturation markers and the co-stimulatory 41BBL and 41BB molecules among all three cDC1 subsets in TDLNs.

### The 41BB-agonist in IRE+Combo ablation triggers potent CD4^+^ Th1 cell responses in TDLNs

**Figure 6:**
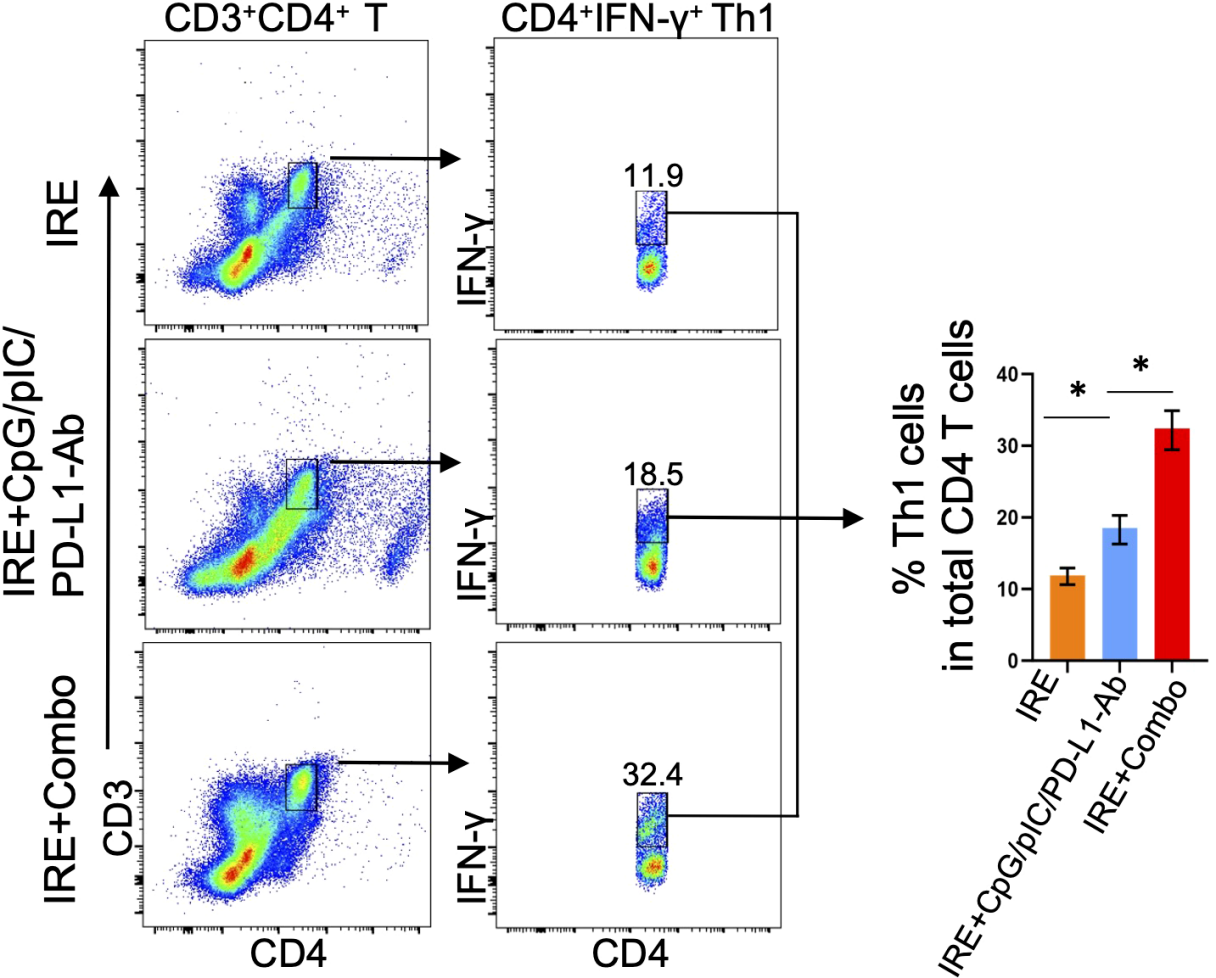
IRE+Combo promotes CD4^+^ Th1 cell responses in TDLNs. Single-cell suspensions prepared from the TDLNs 8 days after primary tumor treatments of IRE, IRE+CpG/pIC/PD-L1-Ab and IRE+Combo, respectively. Cell samples were stained with a cocktail of Abs to analyze expression of CD3, CD4 and IFN-γ by flow cytometry. Flow cytometry plots and bars showed quantitative measurement of the percentage of CD4^+^IFN-γ^+^ Th1 by analysis of gated CD3^+^CD4^+^ T cells in IRE, IRE+CpG/pIC/PD-L1-Ab and IRE+Combo ablation groups, respectively. *P < 0.05 by two-tailed Student *t*-test. The above data represent one of two independent experiments.

It has been reported that CD4^+^ Th1 cells license DCs by using CD40L to interact with DC’s CD40, leading to DC maturation and subsequent stimulation of potent CD8^+^ T cell responses and CD8^+^ T cell memory formation (34, 35). CD4^+^ Th1 cells have also been found to endow cDC1 with cancer-impeding functions and to prevent CD8^+^ T cell exhaustion in the TME (34–37). Therefore, to assess IRE+Combo-induced CD4^+^ Th1 cell responses, we analyzed single cell suspensions prepared from TDLNs by flow cytometry (23). Briefly, a population of CD3^+^CD4^+^ T cells was sorted by flow cytometry to quantitate CD4^+^IFN-γ^+^ Th1 cells (**Figure 6**). We demonstrated that inclusion of the 41BB-agonist in the IRE+Combo treatment triggered a more efficient CD4^+^IFN-γ^+^ Th1 cell response (32.4%) in total CD4^+^ T cells when compared to IRE+CpG/pIC/PD-L1-Ab (18.54%) or IRE (11.9%)-induced Th1 cells (**Figure 6**), indicating that the 41BB-agonist plays an important role in augmenting CD4^+^ Th1 responses in TDLNs.

### The 41BB-agonist in IRE+Combo ablation directly promotes potent 41BB^+^CD103^+^TCF-1^+^ T_RM_ cell responses in TDLNs

**Figure 7:**
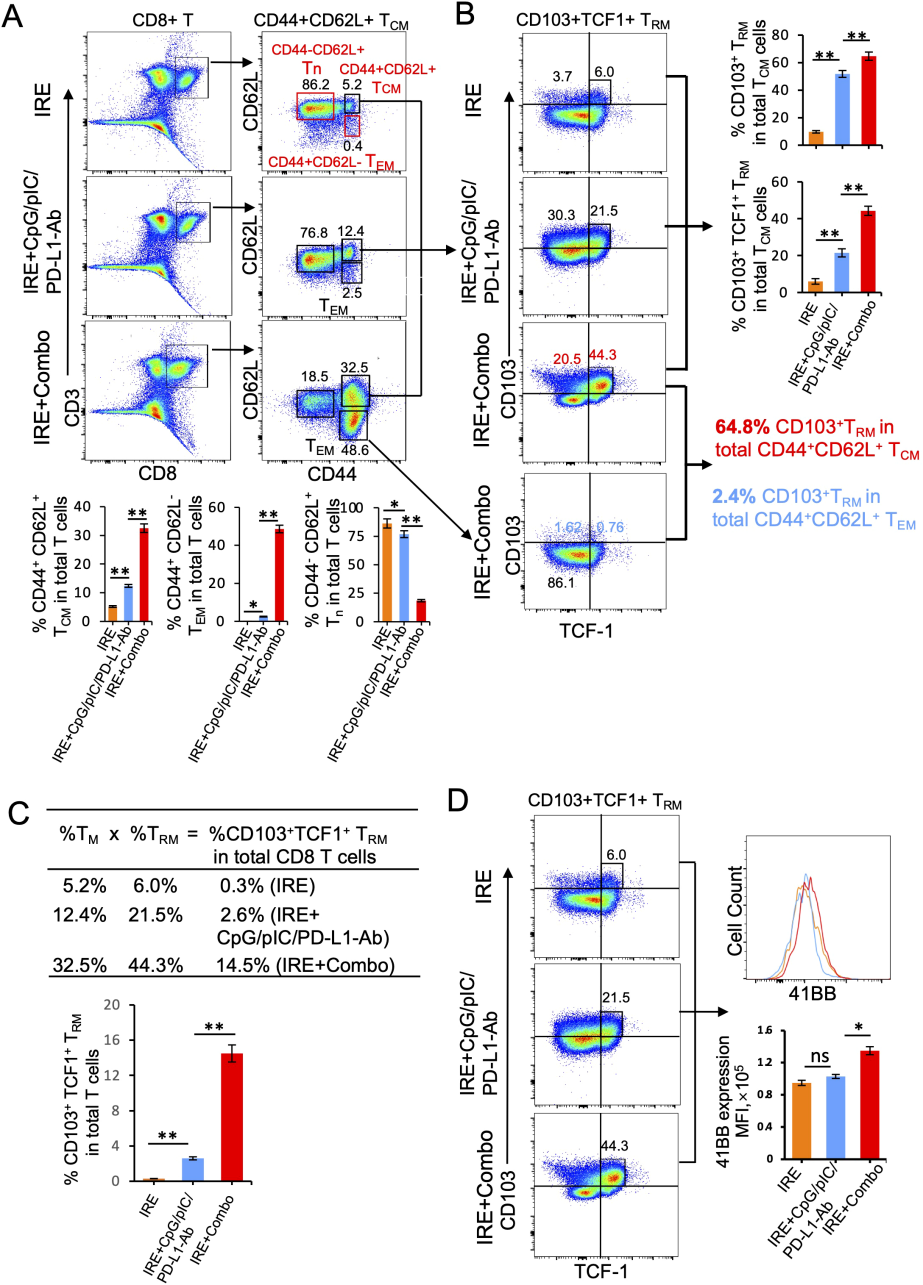
IRE+Combo promotes CD8^+^ T_RM_ cell responses in TDLNs. Single-cell suspensions prepared from the TDLNs 8 days after primary tumor treatments of IRE, IRE+CpG/pIC/PD-L1-Ab and IRE+Combo, respectively. Cell samples were stained with a cocktail of Abs to analyze the expression of CD3, CD8, CD44, CD62L, CD103, TNF-1 and 41BB by flow cytometry, respectively. **(A)** Flow cytometry plots and bars showed quantitative measurement of the percentage of three CD8^+^ T cell subsets (CD44^-^CD62L^+^ Tn, CD44^+^CD62L^-^ T_EM_ and CD44^+^CD62L^+^ T_CM_) by analysis of gated CD3^+^CD8^+^ T cells in IRE, IRE+CpG/pIC/PD-L1-Ab and IRE+Combo groups. **(B)** Flow cytometry plots and bars showed quantitative measurement of the percentage of CD103^+^ T_RM_ and CD103^+^TCF-1^+^ T_RM_ in total of CD44^+^CD62L^+^ T_CM_ cell population by analysis of gated CD44^+^CD62L^+^ T_CM_ cells in IRE, IRE+CpG/pIC/PD-L1-Ab and IRE+Combo groups, respectively. Flow cytometry plots also showed quantitative measurement of the percentage of CD103^+^ T_RM_ in total of CD44^+^CD62L^+^ T_CM_ cell and CD44^+^CD62L^-^ T_EM_ cell population by analysis of gated CD44^+^CD62L^+^ T_CM_ and CD44^+^CD62L^-^ T_EM_ cells in IRE+Combo group, respectively. **(C)** In the table, %T_CM_ (in total CD8^+^ T cell population) × %T_RM_ (in total T_CM_ cells) = %T_RM_ in total CD8^+^ T cells. **(D)** The expression of 41BB on CD103^+^TCF-1^+^ T_RM_ cells in IRE, IRE+CpG/pIC/PD-L1-Ab and IRE+Combo ablation groups were analyzed by flow cytometry, respectively. The values of the MFI of 41BB expression for each group were shown in flow cytometry histogram sets. *P < 0.05, **P < 0.01 by two-tailed Student *t*-test. The above data represent one of two independent experiments.

TDLNs are lymphoid organs that also initiate the anti-tumor immunity of CD8^+^ T cells. In TDLNs, various CD8^+^ T cell subsets are capable of differentiating into CD103^+^ T_RM_ cells. For example, naïve T (Tn), T_CM_ and T_EM_ cells stimulated by stromal cell-produced TGF-β and by BATF3^+^ DC-secreted TGF-β or IL-15 can be further differentiated into CD103^+^ T_RM_ cells (38–40). The cytokines TGF-β and IL-15 can also induce CD8^+^ T_RM_ cell formation in non-lymphoid tissues (31, 41). CD103^+^ T_RM_ cells are often found throughout various peripheral tissues or organs such as the skin, liver, intestine and lung (42, 43). However, CD103^+^ T_RM_ cells can also accumulate and expand in regional lymph nodes in mouse tumor and infection models (44–47). T cell factor-1 (TCF1) is a transcriptional factor that sustains this T_RM_ cell-directed anti-tumor immunity (48). To assess the ability of IRE+Combo treatment to induce CD8^+^CD44^+^CD62L^+^ T_CM_ and CD8^+^CD44^+^CD62L^+^CD103^+^ T_RM_ cell responses, we analyzed TDLN single cell suspensions by flow cytometry with progressive gating strategies as previously described (23). Briefly, a group of CD3^+^CD8^+^ T cells were sorted by flow cytometry to quantitatively analyze CD8^+^ T cell subsets (**Figure 7A**). These analyses revealed three clearly defined CD8^+^ T cell subsets comprised of CD44^+^CD62L^+^ T_CM_, CD44^+^CD62L^-^ T_EM_ and CD44^-^CD62L^+^ Tn cells (49, 50) in the IRE+Combo flow cytometry plots (**Figure 7A**). Although there was not a clearly defined CD44^+^CD62L^-^ T_EM_ cell population in IRE and IRE+CpG/pIC/PD-L1-Ab plots, we used the same gating as the IRE+Combo plot to define these cells (**Figure 7A**). Through these analyses, we demonstrated that inclusion of 41BB-agonist in the IRE+Combo treatment promoted more efficient CD44^+^CD62L^+^ T_CM_ and CD44^+^CD62L^-^ T_EM_ cell responses (32.5% and 48.6%) when compared to the IRE+CpG/pIC/PD-L1-Ab (12.4% and 2.5%) and IRE (5.2% and 0.4%) control treatment groups (**Figure 7A**). We then gated CD44^+^CD62L^+^ T_CM_ cells by flow cytometry to quantitatively analyze CD103^+^TCF-1^+^ T_RM_ cells (**Figure 7B**). Our IRE+Combo flow cytometry plot showed four defined CD8^+^ T cell subpopulations comprised of CD103^+^TCF1^+^ and CD103^+^TCF1^-^ T_RM_ as well as CD103^-^TCF1^-^ and CD103^-^TCF1^+^ T_M_ cells (**Figure 7B**). We then used the same gating parameters from the IRE+Combo plot to define these four CD8^+^ T cell subpopulations in IRE and IRE+CpG/pIC/PD-L1 Ab plots (**Figure 7B**). These analyses demonstrated that a higher frequency of CD103^+^ T_RM_ cells (20.5%+44.3%=64.8%), which included a specific CD103^+^TCF1^+^ T_RM_ (44.3%) cohort, were found in the total CD44^+^CD62L^+^ T_CM_ cell population of IRE+Combo-treated mouse TDLNs compared to the two control treatment groups (30.3 + 21.5 = 51.8% and 3.72 + 6.0 = 9.72% or 21.5% and 6.0%) (**Figure 7B**). To assess CD103^+^ T_RM_ cells derived from CD44^+^CD62L^-^ T_EM_ cells, we gated CD44^+^CD62L^-^ T_EM_ cells accordingly and measured the abundance of CD103^+^ T_RM_ cells by flow cytometry. We demonstrated that unlike their rich representation in the CD44^+^CD62L^+^ T_CM_ cell population, CD103^+^ T_RM_ cells comprised only 2.4% (1.6%+0.8%=2.4%) of the total CD44^+^CD62L^-^ T_EM_ cell population(**Figure 7B**), indicating that a higher frequency of 41BB agonist-promoted CD103^+^ T_RM_ cells are derived from the CD44^+^CD62L^+^ T_CM_ as opposed to CD44^+^CD62L^-^ T_EM_ cell population in this therapeutic context. A higher frequency of CD103^+^TCF1^+^ T_RM_ cells (14.5%) was also observed in the total CD8^+^ T cell population in IRE+Combo-treated mouse TDLNs compared to the two control treatment groups (2.6% and 0.3%) (**Figure 7C**), which indicates that the 41BB-agonist can enhance CD8^+^ T_RM_ cell responses and is consistent with a previous report that 41BB-agonist promoted expansion of tumor-infiltrating CTLs (51). To measure 41BB expression, we further gated CD44^+^CD62L^+^ T_CM_ cells by flow cytometry to analyze T cell co-stimulatory 41BB expression (**Figure 7D**). The representative flow cytometry histograms showed significantly more 41BB expression on IRE+Combo-triggered CD8^+^ T_CM_ cells relative to CD8^+^ T_CM_ cells from the two control treatment groups (**Figure 7D**). Our data thus indicate that the 41BB-agonist plays an important role in upregulating 41BB and TCF1 expression and promoting the expansion of 41BB^+^CD103^+^TCF1^+^ T_RM_ cells.

### IRE+Combo treatment converts the TME by modulating the activity and properties of MDSCs, cDC1 and CD8^+^ T cells

**Figure 8:**
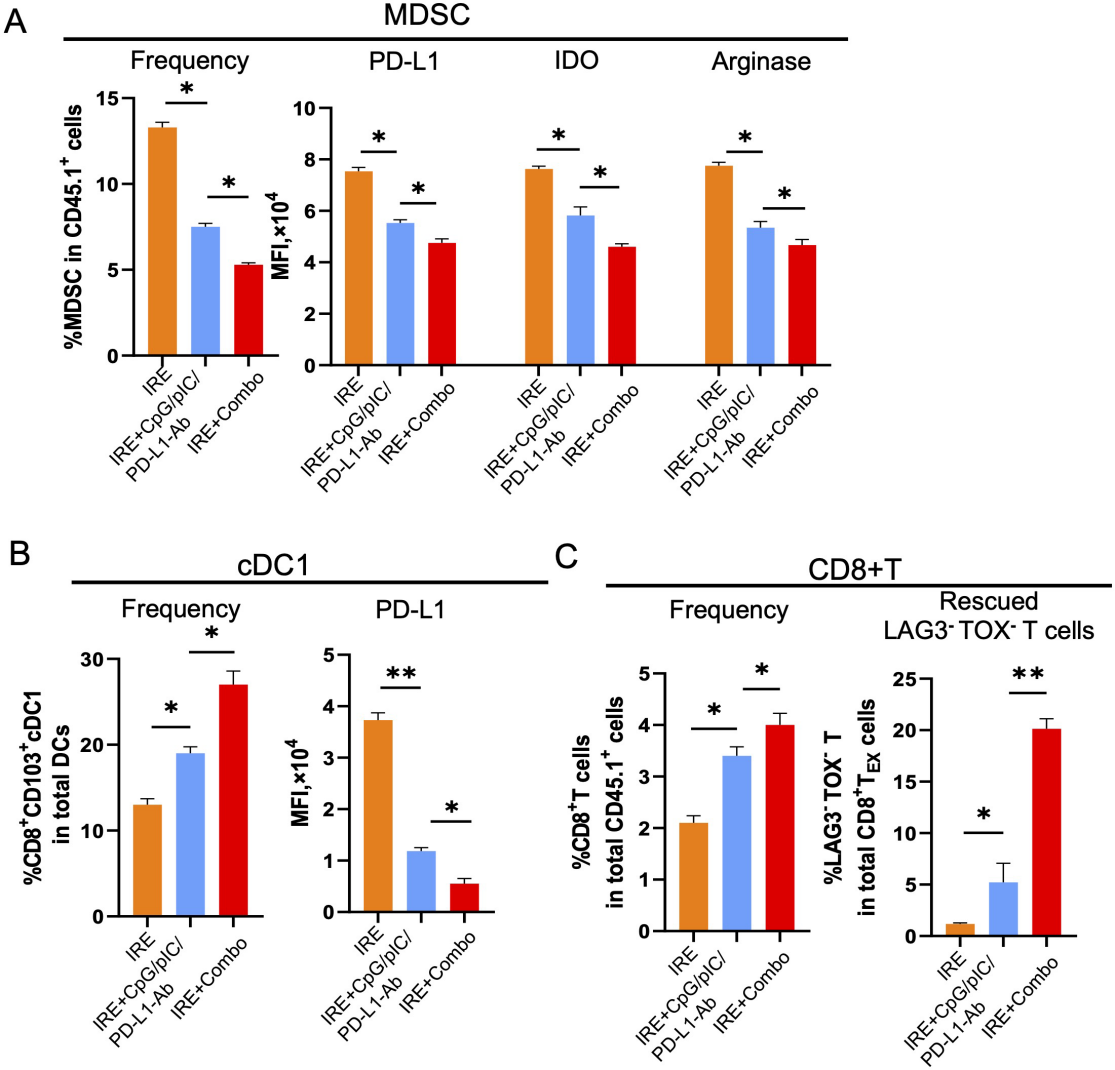
IRE+Combo ablation converts TME by modulation of MDSCs, cDC1 and CD8^+^ T cells. **(A)** Single-cell suspensions were prepared from primary tumor tissues collected from B6.1 mice treated with IRE, IRE+CpG/pIC/PD-L1-Ab and IRE+Combo 3 days post the treatment. Cell samples were stained with a cocktail of Abs against MDSC markers and analyzed by flow cytometry as described in the Methods. The percentage of MDSCs in CD45.1^+^CD11b^+^ was shown as Frequency. The expression of PD-L1, IDO and arginase-1 in gated CD45.1^+^CD11b^+^ was shown as the MFI. **(B)** Cell samples were also stained with a cocktail of Abs for cDC1 markers and analyzed by flow cytometry. The percentage of cDC1 in CD45.1^+^CD11b^+^ was shown as Frequency. The expression PD-L1 in gated CD45.1^+^CD11b^+^ was shown as the MFI. **(C)** Cell samples were permeabilized and stained with a cocktail of Abs for CD8^+^ T cell markers and intracellular LAG3 and TOX and then analyzed by flow cytometry. The percentage of CD8^+^ T cells in total CD45.1^+^ cell population was shown as Frequency. The percentage of rescued CD8^+^LAG3^-^TOX^-^ T cells in total exhausted CD8^+^LAG3^+^TOX^+^ T_EX_ cell population was shown. *P < 0.05, **P < 0.01 by two-tailed Student *t*-test. The above data represent one of two independent experiments.

Having established the potent therapeutic effect of the IRE+Combo treatment, we next investigated its influence on the immune cell profile of the TME. CD11b^+^Gr1^+^Ly6G^+^ MDSCs, the major player or the “Queen Bee” for the TME (52), express the inhibitory molecules PD-L1, arginase-1 and indoleamine 2,3-dioxygenase (IDO) (53). cDC1 cells are capable of cross-presenting antigen and potently stimulating anti-tumor CD8^+^ T cell responses in both TDLNs and tumors (25–27). While CD8^+^ T cells are critical for anti-tumor immunity (54), they often become dysfunctional in the TME and express the exhaustion markers LAG3 and TOX (55). We thus focused on these three important immune cell types of the TME, and analyzed them by flow cytometry using single cell suspensions prepared from IRE-, IRE+CpG/pIC/PD-L1-Ab- and IRE+Combo-treated 3LL_OVA_ tumors 3 days post-ablation as we previously described (23). These analyses demonstrated that the IRE+Combo treatment led to a more significant reduction in the frequency of immunotolerant CD11b^+^Gr1^+^Ly6G^+^ MDSCs (5.0% in CD45.1^+^CD11b^+^ cells) than either the IRE+CpG/pIC/PD-L1-Ab or IRE treatments (7.5% or 13.2% MDSCs in CD45.1^+^CD11b^+^ cells) (**Figure 8A**). Consistent with this observation, the IRE+Combo treatment also more markedly down-regulated expression of inhibitory PD-L1, IDO and arginase-1 in MDSCs when compared with the IRE+CpG/pIC/PD-L1-Ab or IRE alone treatments (**Figure 8A**). In addition, we also found that IRE+Combo significantly promoted the immunogenic potential of cDC1 cells in the TME. Indeed, IRE+Combo treatment markedly increased the frequency of CD8^+^CD103^+^ cDC1 cells while down-regulating their PD-L1 expression when compared with IRE+CpG/pIC/PD-L1-Ab and IRE treatments (**Figure 8B**). We also observed a significant increase in the frequency of CD8^+^ T cells upon IRE+Combo ablation (**Figure 8C**). More importantly, however, IRE+Combo ablation resulted in a much more pronounced increase in the frequency of rescued CD8^+^LAG3^-^TOX^-^ T cells relative to the IRE+CpG/pIC/PD-L1-Ab and IRE alone treatments (**Figure 8C**). In fact, non-exhausted CD8^+^LAG3^-^TOX^-^ T cells represented 20% of the total CD8^+^ T cell population in IRE+Combo treated tumors, which was 4-fold higher than that observed in IRE+CpG/pIC/PD-L1-Ab ablated tumors (**Figure 8C**). Taken together, our data clearly indicate that the 41BB-agonist critically contributes to the potent ability of the IRE+Combo treatment to convert the TME by efficiently modulating the abundance and properties of immunosuppressive MDSCs and immunogenic CD8^+^CD103^+^ cDC1 as well as by significantly rescuing CD8^+^ T cell exhaustion.

## Discussion

Several recent studies have investigated how to improve the therapeutic efficacy of various IRE-ablation protocols in mouse solid cancer models (53, 56–61). For example, Narayana et al. demonstrated IRE-ablation combined with TLR7 agonist/PD-1 blockade (53) or CD40 agonist (56) improved the therapeutic effect on growth inhibition of KPC pancreatic cancer (~5 mm) by modulating the TME. Two other research groups combined IRE-ablation with STING agonist and investigated its therapeutic efficacy in lung cancer (3–4 mm) and melanoma (5 mm) models (57, 58). While this treatment protocol led to the suppression of tumor growth, it did not result in tumor eradication (57, 58). Woeste et al. showed that IRE combined with β-glucan administration significantly prolonged survival of mice bearing KPC pancreatic cancer (3–4 mm) (59). Zhang et al. demonstrated that IRE combined with OX40 agonist resulted in eradication of KPC1199 pancreatic cancer (6–9 mm) in 80% of tumor-bearing mice (60). To assess the therapeutic effect in a given mouse tumor model, a qualitative analysis of tumor-specific CD8^+^ T cell responses provides a digital measurement critical for anti-tumor immunity; however, tumor-specific CD8^+^ T cell responses were not assessed in any of the aforementioned tumor models, possibly because most endogenous TAs were not defined with respect to the generation of their respective Abs. Recently, Burbach et al. investigated the therapeutic effect of IRE-ablation combined with PD-1/CTLA4 blockades in an endogenous TA SPAS-1 (stimulator of prostatic adenocarcinoma-specific T cell-1)-expressing TRAMP-C2 prostate cancer (5–6 mm) model, and found that the combined IRE-ablation protocol stimulated SPAS-1-specific CD8^+^ T cell responses and led to sustained tumor remission but not eradication (61).

OVA has been commonly used as a nominal TA and widely applied to establish mouse OVA-expressing tumor models (23, 24, 62–65). In this study, we established a 3LL_OVA_ mouse model of lung cancer (50 mm^3^) engineered to express the TA transgene OVA. To enhance the therapeutic efficacy of IRE-ablation in 3LL_OVA_ lung cancer, we developed a novel IRE+Combo ablation protocol with the capacity to simultaneously trigger four distinct immune mechanisms. These include pIC-stimulated CD4^+^ Th1 cell responses, CpG-triggered DC maturation leading to enhanced stimulation of CD8^+^ T cell responses (9, 10), PD-1 blockade-induced restoration of T cell exhaustion (14, 15) and 41BB-agonist-promoted CTL expansion, persistence and resistance to T cell exhaustion (17–22). We then investigated its therapeutic efficacy in our 3LL_OVA_ lung cancer model. Interestingly, we demonstrated that addition of the 41BB-agonist to the IRE+CpG/pIC/PD-L1 Ab protocol significantly stimulated potent OVA-specific CD8^+^ T cell responses in peripheral blood and CD8^+^ T_RM_ cell and cDC1 subset responses in TDLNs when compared with all other treatment regimens. Consistent with these findings, the IRE+Combo ablation resulted in the eradication of local s.c. 3LL_OVA_ cancer in 75% of mice and completely eradicated lung BL_OVA_ melanoma metastasis.

**Figure 9:**
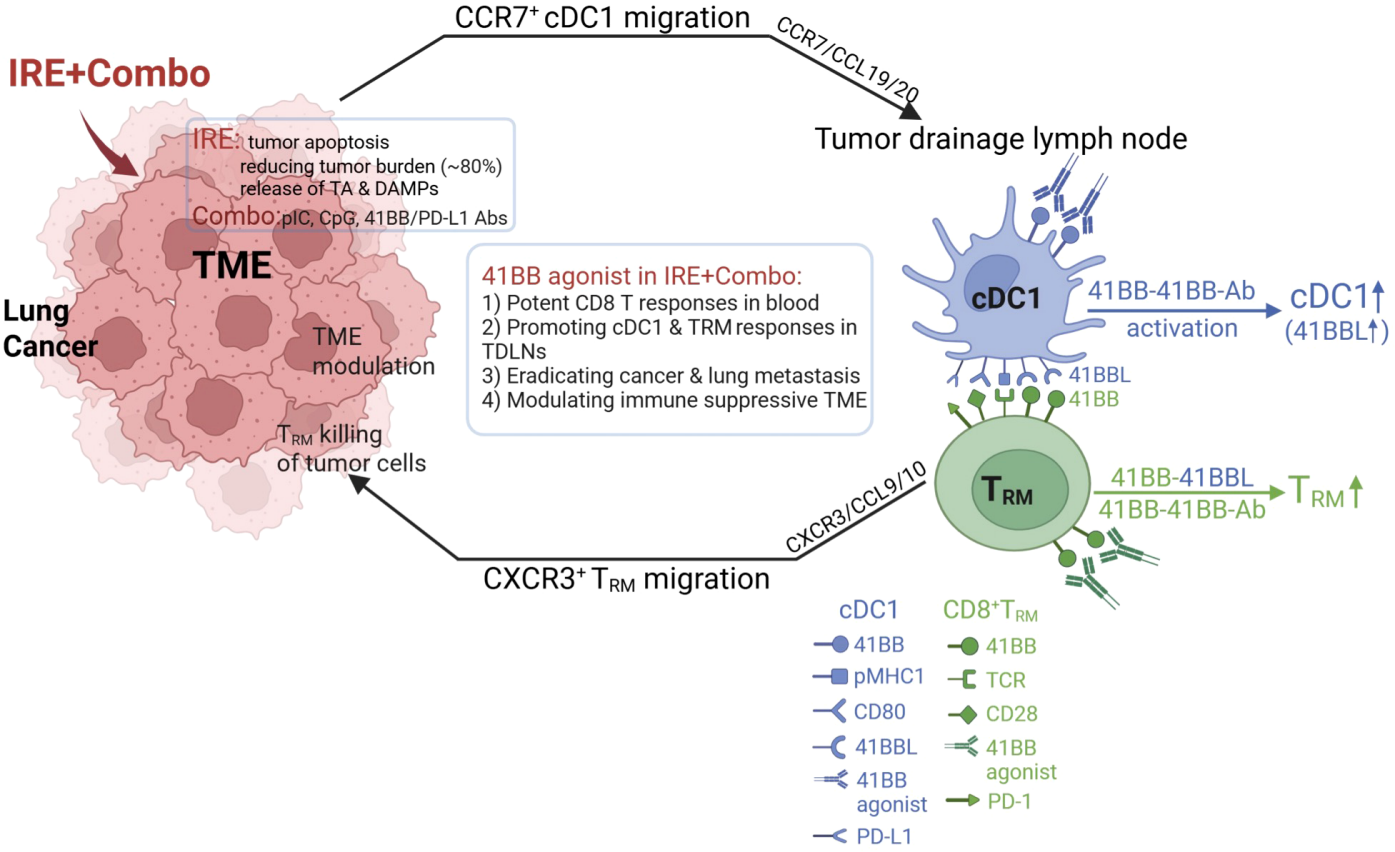
Schematic illustrating how 41BB-agonist signaling in combined IRE+Combo ablation promotes cDC1 and T_RM_ cell responses in TDLNs, leading to conversion of TME and eradication of 3LL_OVA_ cancer and lung tumor metastases.

Cross priming is a process during which CCR7^+^CXCR3^+^ DCs (66, 67) present exogenous antigens to CD8^+^ T cells, leading to T cell activation and expansion. cDC1 is a rare but superior subset of DCs capable of antigen cross-presentation and potent stimulation of CD8^+^ T cell responses in TDLNs (25–27). In tumors, DC and cDC1 that phagocytose apoptotic tumor cell fragments migrate to TDLNs in a CCR7-dependent manner (30), where they cross-present TA to CD8^+^ T_RM_ cells to potentiate their expansion in TDLNs (**Figure 9**). In TDLNs, CXCR3^+^ cDC1 activated by the 41BB-agonist can also migrate into tumors in a CXC3R3/CXCL9/10 manner (**Figure 9**) (66, 67). Furthermore, CXCL9/10-expressing cDC1 and IFN-γ-stimulated tumor cells (68) can also recruit CXCR3^+^ T_RM_ or T_E_ cells into tumors through T cell tumor infiltration (69, 70).

41BB is a T cell co-stimulatory molecule of the tumor necrosis factor receptor (TNFR) family (16, 71). To date, most of its expression has been transiently observed on T cell receptor (TCR)- or CD3-activated T cells (72). The co-stimulatory 41BB-agonist has been found to enhance CD8^+^ T cell proliferation, cytotoxicity and persistence and rescue T cell exhaustion (17–22), thereby potentiating cancer immunotherapy. Triggering the T cell co-stimulatory 41BB signaling of DCs has also been found to increase DC production of IL-12 and promote CD4^+^ Th1 responses (72, 73). In this study, we provide the first evidence that inclusion of the 41BB-agonist in our IRE+Combo treatment regimen promoted potent 41BB^+^CD103^+^TCF1^+^ T_RM_ cell responses above those previously reported to be triggered by intrinsic 41BBL signaling (16). We also demonstrate that adding the 41BB-agonist in our IRE+Combo treatment regimen promoted potent 41BB^+^ cDC1 responses that included expansion of cDC1 subsets and up-regulated expression of the DC maturation markers CD54, CD80 and MHC II and the co-stimulatory DC 41BBL and 41BB T cell molecules. These observations provide a potential mechanistic reason for why the 41BB-agonist can promote robust 41BB^+^CD103^+^TCF1^+^ T_RM_ cell responses in TDLNs whose potency in the TDLNs of IRE-Combo-treated mice likely involve both a direct 41BB/41BB-agonist interaction and an indirect 41BB/cDC1 41BBL interaction (**Figure 9**).

The transcription factor TCF1 is a significant biomarker that plays an important role in T cell development and stem-cell like differentiation (74). TCF1 also improves the effect of PD-1 blockade (75) and sustainment of anti-tumor CD8^+^ T cell responses (43, 76). Therefore, TCF1 expression has become a significant biomarker of stem-cell like properties associated with improved anti-tumor immunity and alleviated CD8^+^ T cell exhaustion in the TME (77). CD8^+^CD103^+^TCF1^+^ T_RM_ cells exhibited much stronger cytotoxicity towards tumor cells than conventional CD8^+^ T_E_ cells (78, 79). Therefore, CD8^+^CD103^+^TCF1^+^ T_RM_ cell responses become a biomarker for the prognosis of cancer patients (78, 79).

In addition to CXCL9/10-expressing cDC1, T_RM_ cell recruitment to, and infiltration of, tumors (68) is facilitated by T_RM_ cell expression of CD103 which allows for adhesion to the cadherin of tumor cells (80, 81). CD103 is then recruited into the immune synapse where it promotes exocytosis of oncolytic granules containing perforin and granzyme B, which leads to the lysis of tumor cells (**Figure 9**) (82, 83). Considering the reduced mass of tumor tissues and the less suppressive TME following IRE+Combo ablation, tumor-infiltrated CD8^+^CD103^+^TCF1^+^ T_RM_ cells should exhibit prolonged survival, greater resistance to exhaustion and more cytotoxicity to tumor cells, leading to complete eradication of IRE+Combo-treated 3LL_OVA_ lung cancer (**Figure 9**).

The TME has become a major obstacle in immunotherapy of solid cancers such as lung cancer, and is infiltrated by many immunosuppressive immune cells (84). These include inhibitory type 2 macrophage (M2), MDSCs and CD4^+^ regulatory T (Treg) cells expressing suppressive PD-L1 and secreting the inhibitory cytokines TGF-β and IL-10 (23, 84). To elucidate the mechanism underlying IRE+Combo-induced eradication of s.c. 3LL_OVA_ lung cancer, we also analyzed single cell suspensions enzymatically prepared from IRE-, IRE+CpG/pIC/PD-L1 Ab- and IRE+Combo-treated 3LL_OVA_ cancer tissues by flow cytometry, as we previously described (23). We demonstrated that IRE+Combo converted the immunosuppressive TME of 3LL_OVA_ lung cancer by (i) decreasing the frequency of inhibitory MDSCs as well as down-regulating their production of inhibitory PD-L1, IDO and arginase; (ii) increasing the frequency of immunogenic CD8^+^CD103^+^ cDC1 and down-regulating the abundance of inhibitory cell surface PD-L1; and (iii) rescuing CD8^+^ T cell exhaustion. The reversal of CD8^+^ T cell exhaustion in TME of IRE+Combo-ablated 3LL_OVA_ lung cancer may reflect a cumulative effect of (i) PD-1 blockade (13, 14), (ii) endowment of cDC1 with cancer-impeding functions by CD4^+^ Th1 cells derived from TLR3 agonist (pIC) stimulation (34, 35), and (iii) the alleviating effects of CD103^+^TCF1^+^ T_RM_ cells (77).

The 41BB-agonists have long been applied to human cancer immunotherapy and have shown significant clinical activity (85). Combination of 41BB-agonist with PD-1 blockade induced durable, potent anti-tumor effector/memory T cell responses in animal tumor models (86, 87). In the clinic, the human CD137/PD-L1 bi-specific Ab has shown its enhanced anti-tumor immune responses through activation of tumor-specific T cells and immune checkpoint blockade (88–90). However, hepatotoxicity derived from strong 41BB stimulation restrains its clinic utility. To minimize Fcγ-receptor cross-linking-induced hepatotoxicity (85), a new wave of 41BB-targeted, engineered bi-specific antibodies with a modified Fc that retains the 41BB-stimulating arm while adding selectivity for tumors to reduce unwanted side effects have been applied in clinical trials. For example, the recently developed bi-specific antibodies GEN1046 and MCLA-145 targeting both 41BB and PD-L1 have shown promising safety in the clinic (91).

## Conclusion

Taken together, our results showed that the IRE+Combo is a promising, novel therapeutic protocol that combines IRE-ablation with immune adjuvant Combo to boost superior cDC1 and powerful CD8^+^ T_RM_ cell responses against lung cancer. We demonstrated that (i) IRE-ablation was capable of reducing lung cancer burden through its efficient induction of tumor cell apoptosis and the release of large amounts of TAs and DAMP adjuvants, and (ii) 41BB-agonist signaling in Combo potentiates the therapeutic efficacy of IRE+Combo ablation in eradicating local s.c. 3LL_OVA_ cancer and lung BL_OVA_ metastasis by promoting responses from CD8^+^, CD103^+^ and unexpected CD8^+^CD103^+^ cDC1 subsets as well as CD103^+^TCF1^+^ T_RM_ cells in TDLNs (**Figure 9**). The powerful CD103^+^TCF1^+^ T_RM_ cells then mediate efficient tumor eradication through secretion of the T cell effector cytolytic granules perforin and granzyme-B after their infiltration into the remaining tumor tissues (in peripheral areas of tumor masses) with IRE+Combo-induced TME modulation (**Figure 9**). This study therefore establishes that the 41BB-agonist potentiates the efficacy of IRE+Combo-therapy for lung cancer treatment by promoting both T_RM_ and cDC1 responses, and emphasizes the importance of targeting this promising molecular signal to improve current IRE-ablation protocols for lung cancer or other solid malignancies such as liver, colon and stomach cancers.

## Data Availability

The original contributions presented in the study are included in the article/supplementary material. Further inquiries can be directed to the corresponding authors.
